# Successful Surgical Treatment of Pediatric Intestinal Behçet’s Disease with Ileocecal Ulcer Refractory to Medical Therapy: A Case Report

**DOI:** 10.70352/scrj.cr.25-0758

**Published:** 2026-04-21

**Authors:** Kazuki Nagashima, Yuki Mizuno, Futaba Miyaoka, Hitoshi Irabu, Yui Innami, Yuko Kamata, Yoshifumi Ito, Kentaro Okamoto, Masaki Shimizu, Yosuke Tanaka, Kenichi Ohashi, Tomoyuki Aruga

**Affiliations:** 1 Department of Surgical Specialties, Pediatric Surgery, Institute of Science Tokyo, Tokyo, Japan; 2 Department of Pediatrics and Developmental Biology, Institute of Science Tokyo, Tokyo, Japan; 3 Department of Pathology, Institute of Science Tokyo, Tokyo, Japan; 4 Department of Surgical Specialties, Institute of Science Tokyo, Tokyo, Japan

**Keywords:** intestinal Behçet’s disease, pediatric surgery, colectomy

## Abstract

**INTRODUCTION:**

High postoperative recurrence and reoperation rates following surgical treatment for intestinal Behçet’s disease (BD) have been reported in adults. In pediatric patients, reports on surgical treatment and clinical course are extremely rare.

**CASE PRESENTATION:**

An 11-year-old girl was diagnosed with intestinal BD with an ileocecal ulcer at the age of 2 and had been maintained on medical therapy. At the age of 8, she was hospitalized due to exacerbation of the intestinal lesions. Despite adjustment of immunosuppressive agents and other medical management, her condition did not improve. One month after admission, she developed a fever of 40°C, vomiting, and elevated inflammatory markers. Contrast-enhanced CT revealed marked wall thickening from the ileocecal region to the transverse colon, accompanied by intramural abscess formation and surrounding inflammation. She was diagnosed with a refractory ileocecal ulcer resistant to medical therapy, and laparoscopic right hemicolectomy with stoma creation was performed. Her postoperative course was uneventful, and she was discharged. As she remained in remission without recurrence, stoma closure was performed 17 months after the initial surgery following steroid tapering. She has remained relapse-free for approximately 3 years since the first operation.

**CONCLUSIONS:**

In this case, postoperative recurrence was a substantial concern. However, surgical intervention ultimately led to marked improvement of the disease, and the patient was able to maintain a more stable daily life compared with the preoperative period. This case represents a valuable example in which surgical treatment was remarkably effective for pediatric intestinal BD.

## Abbreviations

AZP
: azathioprine
BD
: Behçet’s disease
COL
: colchicine
CyA
: cyclosporine
GI
: gastrointestinal
GOL
: golimumab
IBD
: inflammatory bowel diseases
IVIg
: intravenous immunoglobulin
JAK
: Janus kinase
PSL
: prednisolone
VEO
: very early-onset

## INTRODUCTION

Surgical treatment for intestinal BD in adults has been reported to be associated with high postoperative recurrence and reoperation rates, and it is generally reserved for cases refractory to medical therapy or complicated by GI perforation.^1,2)^ In children, however, reports of surgical management are extremely rare, so long-term outcomes remain unclear, making the decision for surgical indication even more challenging. Here, we present a pediatric case of intestinal BD in which surgical treatment was successful, and no relapse has been observed during more than 3 years of follow-up.

## CASE PRESENTATION

A 2-year-old girl presented with abdominal pain, fever, and aphthous stomatitis. A positive pathergy test and colonoscopy revealing multiple cecal-to-transverse colon ulcers established the diagnosis of intestinal BD. Initial therapy included PSL, COL, infliximab, and methotrexate. The clinical course was relapsing–remitting, requiring plasma adsorption therapy before initial discharge. Subsequent relapses at ages 3 and 4 necessitated re-hospitalizations and extensive adjustments of immunosuppressants (mycophenolate mofetil, tacrolimus, CyA, AZP), biologics (adalimumab, GOL), IVIg, and JAK inhibitors.

**Fig. 1:**
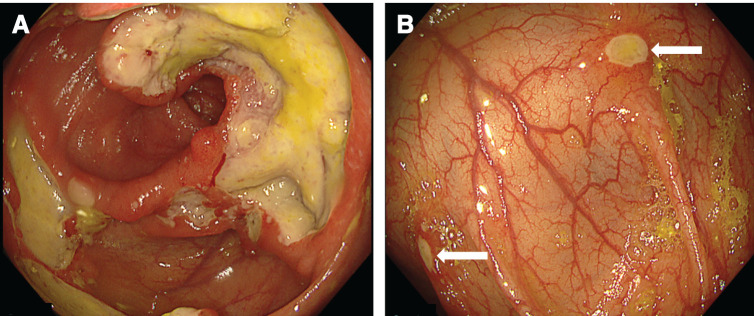
Colonoscopy on hospital day 8. Colonoscopy revealed (**A**) a deep ulcerative lesion with whitish exudate and raised margins involving up to three-fourths of the circumference in the ileocecal region, and (**B**) multiple small ulcerative lesions scattered from the ascending colon to the mid-transverse colon (white arrow).

At age 8, the patient was urgently admitted with abdominal pain, anorexia, low-grade fever, and elevated inflammatory markers. Admission medications included PSL (20 mg/day), GOL, COL, AZP, CyA, and 5-aminosalicylic acid. Colonoscopy revealed a deep, fibrinous, edematous ulcer involving three-fourths of the ileocecal circumference, alongside multiple small ulcers from the ascending to mid-transverse colon (**Fig. 1**). Despite increased PSL, additional IVIg, and antibiotics (cefotaxime, cefmetazole), her condition remained refractory, leading to a 5-kg weight loss.

**Fig. 2:**
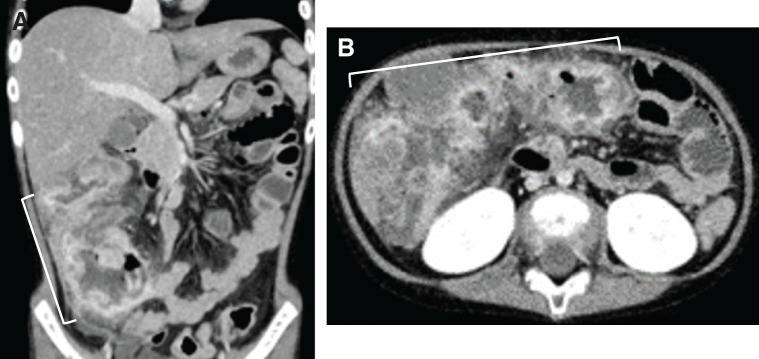
Abdominal CT scans on hospital day 38. On day 38, when the CRP level was markedly elevated, the scans demonstrated mural thickening, intramural abscesses, and increased attenuation of the surrounding fat extending from (**A**) the ileum to (**B**) the transverse colon (bracket). CRP, C-reactive protein

One month after admission, she developed a high fever of 40°C, worsening abdominal pain, and a rapid increase in inflammatory markers. Contrast-enhanced CT revealed marked wall thickening extending from the ileocecal region to the transverse colon, with intramural abscess formation and increased fat stranding in the surrounding adipose tissue (**Fig. 2**). Based on the diagnosis of medically refractory intestinal BD, we elected to proceed with laparoscopic surgery.

**Fig. 3:**
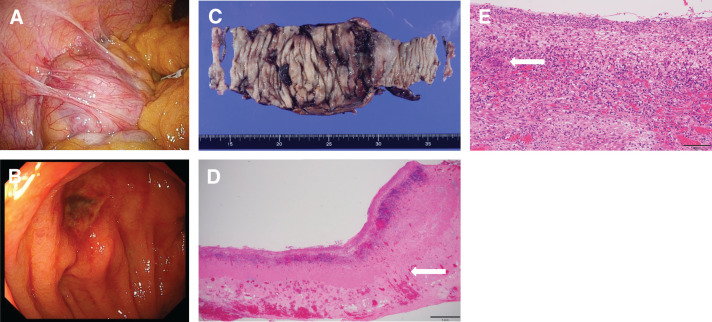
Surgical findings and postoperative pathology. (**A**) Intraoperative laparoscopic view demonstrating mild adhesions around the ascending colon. (**B**) Intraoperative endoscopic view revealing multiple small ulcers extending from the ascending colon to the right side of the transverse colon. (**C**) Resected specimen. (**D**) Histopathological section showing a deep ulcer extending to the subserosal layer with disruption of the muscularis propria (white arrow). (**E**) Granulation tissue characterized by lymphocyte-predominant inflammatory cell infiltration admixed with neutrophils (white arrow).

Trocars were inserted into the umbilicus and right upper abdomen, and laparoscopic inspection revealed inflammatory wall thickening of the ascending colon with mild adhesions (**Fig. 3A**). Additional trocars were placed in both lower quadrants, and the ascending colon was mobilized laparoscopically from the retroperitoneum. A small laparotomy was then made in the right upper abdomen, and the intestine from the terminal ileum to the transverse colon was exteriorized. After appendectomy, intraoperative endoscopy was performed by inserting the endoscope through the cecum via the appendiceal resection stump. The surgeon guided the scope within the operative field, while a separate endoscopist operated the scope from outside the sterile field. This intraoperative endoscopy revealed, in addition to the main ulcer at the ileocecal region, multiple small ulcers extending from the ascending colon to the right side of the transverse colon (**Fig. 3B**). As the absence of ulcerative lesions in the descending colon, sigmoid colon and rectum had been confirmed by a previous endoscopy during the present hospitalization, the observation range was limited to the ascending and transverse colon. The appendiceal stump was re-sterilized and closed, and the surgical procedure was resumed. To ensure complete resection of ulcerative lesions, a right hemicolectomy was performed up to the mid-transverse colon, followed by creation of a double-barrel stoma. Histopathological examination of the resected specimen demonstrated a deep ulcer extending to the subserosal layer with disruption of the muscularis propria. Granulation tissue with lymphocyte-predominant inflammatory cell infiltration admixed with neutrophils was also observed (**Fig. 3C**–**3E**).

**Fig. 4:**
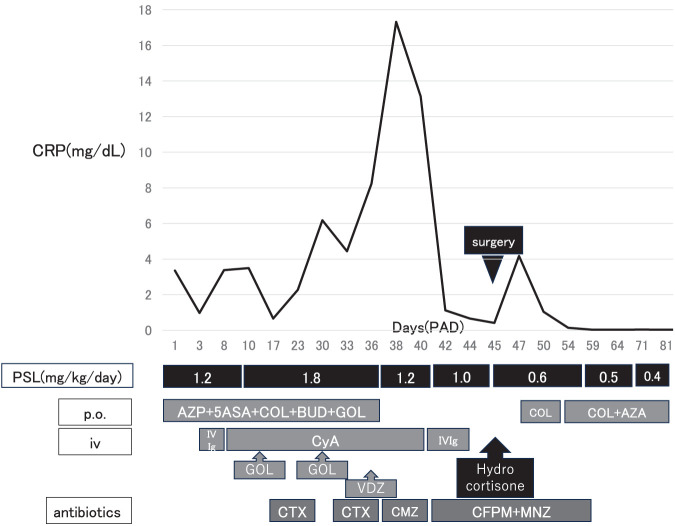
Clinical course of inflammatory markers and medical treatments during hospitalization. 5ASA, 5-aminosalicylic acid; AZP, azathioprine; BUD, budesonide; CFPM, cefepime; CMZ, cefmetazole; COL, colchicine; CRP, C-reactive protein; CTX, cefotaxime; CyA, cyclosporine A; GOL, golimumab; IV, intravenous; IVIg, intravenous immunoglobulin; MNZ, metronidazole; PAD, post-admission day; p.o., per os; PSL, prednisolone; VDZ, vedolizumab

Oral intake was initiated on the first POD, and COL was resumed along with enteral nutrition on POD 2. Solid food intake was restarted on POD 5, and AZP was reintroduced on POD 7. The postoperative course was uneventful, and after adjustment of medications and rehabilitation, the patient was discharged on POD 55 (**Fig. 4**).

**Fig. 5:**
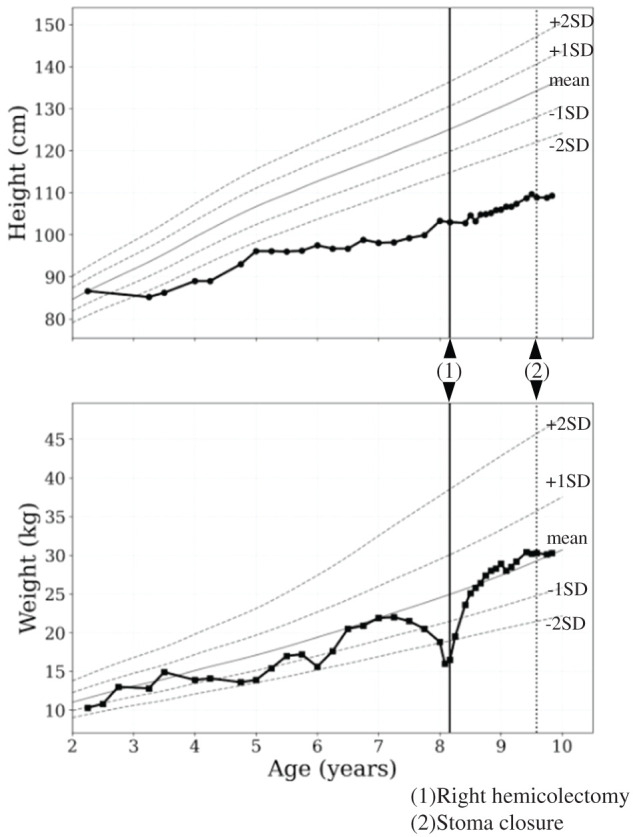
Growth chart (Japanese girls standard). Preoperatively, the patient experienced weight loss due to poor control of BD and difficulty with oral intake. Postoperatively, her clinical course was favorable, and she has since achieved catch-up growth, with her body weight now returning to the same level as before surgery. BD, Behçet’s disease

After discharge, her body weight showed catch-up growth and returned to the preoperative level (**Fig. 5**). As she remained relapse-free, steroids were gradually tapered to 0.2 mg/kg/day of PSL. One year and 4 months after the initial surgery, follow-up colonoscopy confirmed the absence of ulcers or inflammation in the remaining colon and small intestine. One month later, stoma closure was carried out. Approximately 2 cm of bowel was resected on both the colonic and ileal sides, and end-to-end anastomosis was performed using hand-sewn Albert–Lembert sutures with absorbable thread.

Postoperatively, the patient developed fever and markedly elevated inflammatory markers. Contrast-enhanced CT showed no anastomotic leakage or intra-abdominal abscess. Because she remained clinically stable and asymptomatic—without abdominal pain, diarrhea, or vomiting—the episode was attributed to an acute BD exacerbation rather than a surgical complication. She responded well to methylprednisolone pulse therapy. Oral intake and medications were resumed on POD 4, followed by discharge on POD 16. She has remained free of GI relapse for 3 years after surgery, currently maintained on PSL (3.5 mg/day), GOL (50 mg/2 weeks), COL (0.25 mg/day), AZP (50 mg/day), and apremilast (30 mg/day).

## DISCUSSION

BD is a chronic inflammatory disorder characterized by oral, skin, ocular, and genital lesions.^3,4)^ Intestinal BD, a subtype presenting with GI symptoms and ulcers—predominantly in the ileocecal region^5,6)^ lacks specific diagnostic criteria. Diagnosis thus relies on identifying GI lesions in patients meeting general BD criteria. This remains challenging in pediatric cases, as early-stage patients often do not fully satisfy these criteria.^7–9)^

Although BD is more prevalent in East Asia,^10)^ its incidence remains low, estimated at 1–10 per 10000 individuals. GI involvement occurs in only 5%–50% of cases, making intestinal BD a rare manifestation.^11–13)^ Pediatric-onset cases account for 5.4%–7.6% of all BD patients,^14)^ and approximately 50% of these children present with GI symptoms, suggesting a higher frequency of intestinal involvement compared with adult-onset cases.^15)^ Among IBD associated with chronic inflammation, VEO-IBD—defined as disease onset before 6 years of age, as in the present case—accounts for approximately 15% of cases.^16)^ The early onset necessitated excluding monogenic VEO-IBD and autoinflammatory syndromes.^17)^ In this case, we observed no evidence of myelodysplastic syndrome with trisomy 8,^5)^ or A20 haploinsufficiency (TNFAIP3 mutations).^18)^ Furthermore, whole-exome sequencing identified no pathogenic variants in BD susceptibility loci, including IL10 or IL23R–IL12RB2.^5)^ Despite not fully satisfying diagnostic criteria, the patient’s recurrent oral ulcers, positive pathergy reaction, and ileocecal lesions led to a diagnosis of intestinal BD—a common scenario in pediatric cases where major manifestations are often absent.^19)^

The treatment strategy for pediatric intestinal BD has not been fully established,^20)^ and current management is largely based on treatment protocols for other IBD or adult patients. However, pediatric-specific considerations are necessary, such as minimizing the use of corticosteroids due to potential effects on growth. Accordingly, medical therapy was initiated with careful attention to these considerations.

Surgical intervention is indicated for complications—such as perforation, abscess, or bleeding—or medically refractory disease,^21,22)^ typically defined as a steroid-dependent course despite combined immunosuppressive and biologic therapy. However, surgical outcomes for intestinal BD are often poor: approximately 50% of patients experience recurrence within 2 years,^23)^ and the 10-year cumulative reoperation rate reaches 58%.^1)^ Recurrence occurs predominantly (70%) near the anastomotic site.^1)^ Furthermore, postoperative complications including leakage, abscess, and fistula affect ~30% of cases,^24)^ likely reflecting the underlying systemic vasculitis characteristic of BD.^24)^

Reports of surgical treatment for intestinal BD in pediatric patients are extremely rare, and the long-term postoperative outcomes remain unclear. A PubMed search identified only 2 relevant case reports. Zhang and Wang described an 8-year-old Chinese girl with BD who developed multiple intestinal perforations; laparoscopic repair was performed, but an ileocecal perforation occurred 1.5 years later, necessitating an ileocolectomy.^25)^ The second report from Italy described a 12-year-old girl who underwent terminal ileal resection for ileal perforation, with no intestinal recurrence observed during two years of follow-up.^26)^ There are no reports of surgery performed for intestinal BD presenting as VEO-IBD, as in our case. These findings highlight postoperative recurrence and complications as major concerns in our case.

The disease followed a steroid-dependent course, remaining refractory to combined immunosuppressive and biologic therapy. Despite aggressive treatment, poorly controlled disease activity led to progressive weight loss and infectious complications. Given the clinical deterioration and CT findings suggestive of impending ileocecal perforation, urgent surgical intervention was indicated. Following consultation with external pediatric IBD specialists, a colectomy was performed. Histopathological examination of the resected specimen revealed ulcers extending to the subserosa, confirming that the patient was on the verge of perforation and that surgery was unavoidable.

Previous reports have suggested that the use of intraoperative endoscopy during surgical treatment for intestinal BD is associated with lower postoperative recurrence rates compared to procedures without its use, highlighting its clinical usefulness.^2,11)^ Therefore, we used intraoperative endoscopy to determine the extent of disease and performed resection up to the mid-transverse colon to ensure complete removal of ulcerated lesions. Intraoperative endoscopy enabled direct visualization of the residual bowel to detect subtle, discontinuous, or proximally located ulcers, thereby helping to avoid leaving behind active disease that could serve as a nidus for early postoperative recurrence.

In the present case, a primary anastomosis was avoided, and a temporary diverting stoma was created for several reasons. First, the patient was receiving high-dose systemic corticosteroids, which are known to increase the risk of anastomotic leakage. Second, active inflammation persisted at the time of surgery, a condition associated with impaired wound healing and a higher risk of disease recurrence.^27)^ Finally, postoperative recurrence in intestinal BD has been reported to occur frequently at or near the anastomotic site.^1)^ Given these factors, we selected a temporary stoma as the safer surgical approach.

Adequate time was allowed for tapering corticosteroids and adjusting medications to optimize the patient’s general condition before stoma closure. At the time of stoma closure, a hand-sewn end-to-end anastomosis with absorbable sutures was performed to minimize intestinal irritation from residual foreign material. Postoperatively, immunosuppressive agents and corticosteroids were resumed early to control inflammation. Although the individual effects of these interventions remain uncertain, no surgical complications occurred, and GI ulcers did not recur over two and a half years following the initial surgery. Throughout the long disease course since onset at 2 years of age, postoperative status remained particularly stable, and corticosteroids were successfully tapered. These findings suggest that colectomy may be an effective therapeutic option for pediatric intestinal BD that is resistant to medical therapy.

## CONCLUSIONS

We experienced a pediatric case of medically refractory intestinal BD in which surgical intervention was effective. The patient has remained free of recurrence for 3 years postoperatively. These findings suggest that colectomy facilitated by intraoperative endoscopy may represent a viable treatment option for refractory GI ulcers in pediatric BD.

## DECLARATIONS

### Availability of data and materials

Deidentified patient data are available from the corresponding author upon reasonable request and with patient consent.
